# Microbial Therapeutics Designed for Infant Health

**DOI:** 10.3389/fnut.2017.00048

**Published:** 2017-10-26

**Authors:** Claire Watkins, Catherine Stanton, C. Anthony Ryan, R. Paul Ross

**Affiliations:** ^1^APC Microbiome Institute, University College Cork, Cork, Ireland; ^2^Teagasc Food Research Centre, Fermoy, Ireland; ^3^School of Microbiology, University College Cork, Cork, Ireland; ^4^Department of Neonatology, Cork University Maternity Hospital, Cork, Ireland; ^5^School of Science, Engineering and Food Science, University College Cork, Cork, Ireland

**Keywords:** probiotics, prebiotics, gut microbiota, infant, health

## Abstract

Acknowledgment of the gut microbiome as a vital asset to health has led to multiple studies attempting to elucidate its mechanisms of action. During the first year of life, many factors can cause fluctuation in the developing gut microbiome. Host genetics, maternal health status, mode of delivery, gestational age, feeding regime, and perinatal antibiotic usage, are known factors which can influence the development of the infant gut microbiome. Thus, the microbiome of vaginally born, exclusively breastfed infants at term, with no previous exposure to antibiotics, either directly or indirectly from the mother, is to be considered the “gold standard.” Moreover, the use of prebiotics as an aid for the development of a healthy gut microbiome is equally as important in maintaining gut homeostasis. Breastmilk, a natural prebiotic source, provides optimal active ingredients for the growth of beneficial microbial species. However, early life disorders such as necrotising enterocolitis, childhood obesity, and even autism have been associated with an altered/disturbed gut microbiome. Subsequently, microbial therapies have been introduced, in addition to suitable prebiotic ingredients, which when administered, may aid in the prevention of a microbial disturbance in the gastrointestinal tract. The aim of this mini-review is to highlight the beneficial effects of different probiotic and prebiotic treatments in early life, with particular emphasis on the different conditions which negatively impact microbial colonisation at birth.

## Introduction

**Figure 1:**
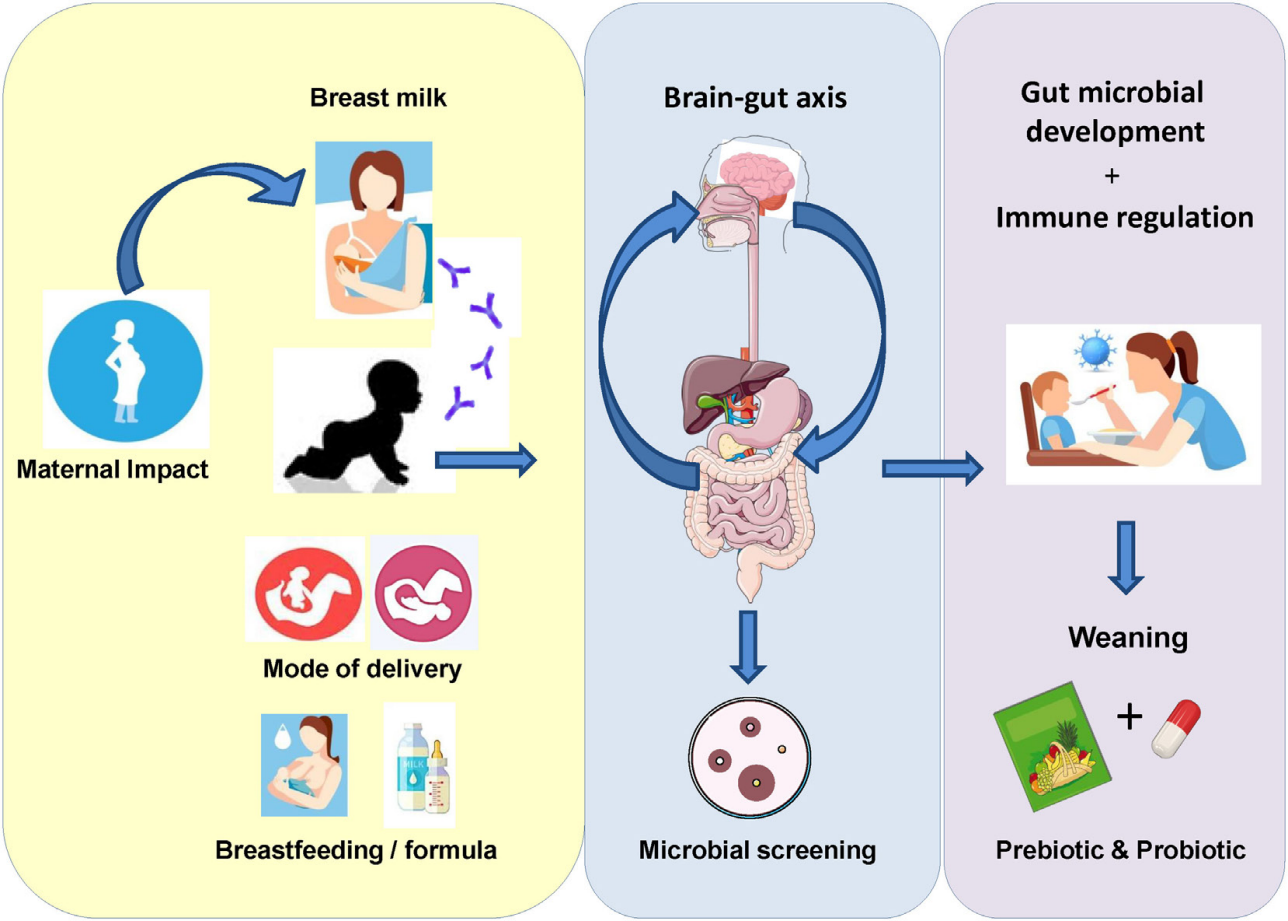
Initial exposure to the microbial environment surrounding the infant can have a significant impact on gut microbiota development. External factors, such as maternal health status, mode of delivery, gestational age, and feeding regime, can impact the colonization and flux of microorganisms during this critical period in life. Subsequently, multiple studies have begun to focus on how these factors can affect the gut microbiome in early life. Moreover, in order to improve the health status of the infant gut, current focus is on the effect of probiotics and prebiotics in terms of their potential multifaceted health benefits. The current mini-review outlines a number of studies where either pro- or pre-biotics were utilized as a microbial therapeutic to improve infant health.

From birth through to the initial stages of weaning, intestinal microbial composition has a significant impact on infant gut health. Recent advances in culture-independent sequencing technologies has allowed for the identification of key microbial species involved in the initial colonization process, including those facultative anaerobes such as *Streptococcus, Staphylococcus*, and *Enterobacter* spp. (1, 2). Mode of delivery and feeding regime are two important factors which influence microbial colonization at birth (Figure 1). Host genetics may also impact development of the gut microbiome, with recent studies focusing on similar microbial patterns between monozygotic twin pairs and their fraternal siblings (3, 4). Indeed, the duration of breast feeding and introduction of formula feed can play a significant role in shaping the gut microbiome (5–7). Thus, it is imperative that we understand how the introduction of particular microbial species and prebiotic additives may restore balance and ameliorate the effects associated with gastrointestinal (GI) disorders.

### GI Microbial Development at Birth

Many studies have begun to focus on the development of the infant gut microbiome over time (8–10). A study by our group found that in full-term (FT) cesarean delivered infants, an increased fecal abundance of Firmicutes and lower abundance of Actinobacteria was evident after the first week of life; however, the gut microbiota of preterm (PT) infants displayed a significantly greater abundance of Proteobacteria compared to the FT infant group. Interestingly, the gut microbiota profile of FT cesarean delivered infants resembled that of the vaginally delivered infants at 8 weeks of life (1). In terms of gestational age at birth, the PT infant gut has previously been characterized by delayed microbial colonization, with reduced levels of anaerobic taxa such as *Bifidobacterium* and *Bacteroides* (11, 12). Indeed, gut microbiota development in PT infants has been found to correlate with the infant’s postconceptual age (13). Moreover, Stewart et al. (14) described the impact of delivery mode on the PT gut microbiome and found no significant change in microbial diversity during the first 100 days of life.

### Maternal–Infant Transmission

The acquisition of microbial strains may occur through multiple different pathways. For example, the administration of the probiotic *Lactobacillus rhamnosus* GG in a small subset of pregnant woman (between 30 and 36 weeks gestational age) found that maternal–infant transmission was successful and identified the strain in the feces of the infant cohort 6 months after birth (15). Indeed, maternal–infant transmission of mothers’ lactobacilli predominantly occurs when the infant is delivered vaginally (16, 17). It is understood that the vaginal tract harbors these lactobacilli to reduce the pH of the intestinal milieu and prevent the growth of potentially pathogenic microorganisms in the infant gut.

Interestingly, a number of studies have begun to examine microbial communities present within breastmilk. Nine genera have previously been identified as part of the “core” breastmilk microbiome, including *Streptococcus, Staphylococcus, Serratia, Pseudomonas, Corynebacteria, Ralstonia, Propionibacterium, Sphingomonas, and Bradyrhizobiaceae* (18). Several other studies have also identified the horizontal transfer of *Lactobacillus, Staphylococcus, Enterococcus*, and *Bifidobacterium* spp., from breastmilk to the infant gut (19–21). In a more recent study, Murphy et al. (22) reported the presence of 12 dominant genera in the breastmilk of lactating mothers. Results from this study described a number of frequently shared taxa, including *Bifidobacterium, Lactobacillus, Staphylococcus*, and *Enterococcus*, common in both breastmilk and infant feces during the first 3 months of life. Moreover, culture-dependent analysis identified *Bifidobacterium breve* and *Lactobacillus plantarum* present in both breastmilk and infant feces. Indeed, similar studies have also identified genomic patterns of *Bifidobacterium* and *Lactobacillus* spp. present in breastmilk and corresponding infant feces (23–25).

Recent studies have also begun to focus on microbial colonization which may occur *in utero*. Isolation of microorganisms from the umbilical cord blood of cesarean delivered infants (26), as well as the detection of bacteria in “first-pass” meconium (27, 28), suggest that the fetus may be colonized at a low abundance prior to exiting the womb. Moreover, research focused on the placental microbiome has found significant correlations between the placental and oral microbial communities (29). However, supporting evidence for the existence of a distinct placental microbiome is currently lacking (30). Thus, microbial colonization of the infant gut may be strongly influenced by the maternal microbiome, originating from several different niches, including the vaginal tract, breastmilk, and possibly the placenta.

Throughout the remainder of this mini-review, a number of studies will be discussed regarding prebiotic and probiotic treatments to prevent and/or treat conditions linked to GI health in early life.

## Prebiotics

### Human Milk Oligosaccharides (HMOs)

The current definition of prebiotics defined by Gibson et al. (31) describes “selectively fermented ingredients that result in specific changes, in the composition and/or activity of the GI microbiota, thus conferring benefit(s) upon host health.”

Human breastmilk is a natural prebiotic source which contains essential nutrients and growth factors required for development of a healthy gut microbiome. Selective proliferation of healthy intestinal bacteria is thought to be just one of the multiple benefits of exclusive breast feeding, in addition to the nutrient supply of HMOs and glycoconjugates it provides (32). As HMOs are not digested by the infant themselves, they reach the colon intact and act as an essential substrate for the growth of beneficial *Bifidobacterium* and *Bacteroides* spp. Breastfed infants have also been found to harbor gut microbial taxa with genes involved in the phosphotransferase system for carbohydrate uptake, in addition to harboring an increased abundance of microbial species commonly used as probiotics, such as *L. johnsonii*/*L. gasseri, L. paracasei/L. casei*, and *B. longum* (33). Moreover, Hill et al. (1) found that prolonged breast feeding (>4 months) had a significant effect on the microbial composition of cesarean delivered FT infants at 24 weeks of life, in comparison to vaginally delivered infants, suggesting that breastmilk may prove to be even more beneficial in caesarian delivered infants.

### Prebiotics and Weaning

It is well known that the infant gut microbiome does not fully develop until an infant reaches 2–3 years of age. Therefore, it is important that we recognize the changes occurring in the infant gut during this transition from early infant feeding to solid foods. Indeed, the World Health Organisation (34) states that the appropriate age for complementary feeding is “6 to 23 months of age”; however, this can change in exceptionally difficult circumstances [e.g., very low birth weight (VLBW) infants]. The following studies investigate the role of diet and the introduction of galacto- and fructo-oligosaccharides (GOS and FOS) for improving gut microbiota development in early life.

In terms of diet, a recent study investigated the impact of different foods on the gut microbiota profile of a Danish infant cohort. Results from this study found strong correlations between microbial taxa present and the dietary intake of foods high in protein and fiber; specifically meats, cheeses, and Danish rye bread (35). Interestingly, breastmilk/early infant feeding was correlated with the presence of *Bifidobacteriaceae, Enterococcaceae*, and *Lactobacillaceae*, whereas *Lachnospiriaceae* abundance was positively correlated with protein intake and negatively correlated with *Bifidobacteriaceae*. Moreover, *Pasteurellaceae* abundance was positively correlated with fiber and health conscious food choices (high in vegetable fats, fruits or fish, but low in sugar). Findings from this study suggest that the transition from breast feeding to “family-like” foods rich in fiber and protein significantly affects development of the infant gut microbiome (35). Digestion of these foods provides a variety of fermentable substrates necessary for the growth of colonic bacteria and thus further investigation into the by-products of predigested foods may provide valuable information to positively modulate the infant gut throughout weaning.

With respect to prebiotic supplementation of infant formulae, recent studies have investigated the use of GOS and FOS to reduce pH and produce a similar short-chain fatty acid (SCFA) profile to that of exclusively breastfed infants (36). Indeed, where infant formulae have been supplemented with GOS/FOS, a higher abundance of *B. longum* was found in the infant gut (37, 38). In addition, Haarman and Knol (38) found that infants consuming a standard formula (without prebiotic supplement) possessed a higher abundance of *Bifidobacterium catenulatum* and *Bifidobacterium adolescentis*, resembling a more adult-like microbiota. Alternatively, prebiotic inulin-type fructans and FOS can be found readily available in foods such as cereals, chicory, and bananas, which are recommended for infants during weaning. These previously mentioned studies, and others (Table 1), provide evidence for the beneficial use of prebiotics, GOS, and FOS, to help maintain a well-balanced microbial progression from infancy to early adulthood.

**Table 1:** (A) Prebiotics effective in altering the intestinal microbiota in human infant studies, (B) probiotic strains effective in altering the intestinal microbiota in a number of human infant studies, and (C) synbiotics effective in altering the intestinal microbiota in a number of human infant studies.

(A) Infant study	Prebiotic	Duration	Microbial shift	Outcome	Reference
Healthy PT–FF	FOS	2 weeks	↑ *BifidobacteriumBacteroides* ↓ *E. coli*	Improved stool frequency	(39)
Healthy FT–FF	GOS + FOS	4–5 weeks	↓ Clostridia	Improved stool frequency	(40)
Healthy PT + FT–FF	GOS + FOS	24 weeks	↕ *BifidobacteriumClostridium*	Increase in sIgA	(41)
Healthy FT–FF	GOS, beta-palmitate + acidified milk	135 days	↕ *BifidobacteriumClostridium*	Adequate growth. Increasing anthropometric parameters	(42)
Healthy FT–FF	GOS + FOS	6 weeks	↑ *Bifidobacterium*	Increase in acetate, butyrate, propionate. Reduced fecal pH	(43)
Healthy FT (>1 year age)–FF	GOS, FOS + inulin	8 weeks	↕ *BifidobacteriumClostridium perfringens*	Increase in total organic acids. Lactacte, acetate, proprionate, butyrate	(44)

**(B) Infant study**	**Probiotic**	**Duration**	**Microbial shift**	**Outcome**	**Reference**

Healthy FT–FF	*L. rhamnosus* GG	24 weeks	↑Lactobacilli	Increased length and weight. Improved growth	(45)
Low birth weight PT–BF + FF	*Bifidobacteriumbreve, Bifidobacteriumlongum* ssp.*infantis,B. longum* ssp.*longum*	6 weeks	↑ *Bifidobacterium↓ ClostridiumEnterobacteriaceae*	Promoted the formation of a healthy gut microbiota. *B. breve* suggested more suitable for PT infants	(46)
Late PT infants–FF	*Clostridium butyricum, Bifidobacterium*	1 week	Not reported	Proliferation of T lymphocytes.Clinical evaluation for *C. butyricum*	(47)
Healthy FT–FF	*Bifidobacterium animalis* ssp.*lactis, Streptococcus thermophiles*.	~1 year	Not reported	Lower frequency of reported colic or irritability.Lower frequency of antibiotic use	(48)

**(C) Infant study**	**Synbiotic**	**Duration**	**Microbial shift**	**Outcome**	**Reference**

Healthy FT–FF	*B. animalis* ssp. *lactis* + bovine milk oligosaccharide	48 weeks	↑ *BifidobacteriumLactobacillus* *↓ClostridiumStaphylococcus*	Supports normal growth. Fecal IgA and pH similar to breastfed infant	(49)
Healthy FT–FF	*B. animalis* ssp. *lactis* + bovine milk oligosaccharide	24 weeks	↑ *Bifidobacterium*	No differences in anthropometric measurements. Lower fecal pH	(50)

*↑, increased levels; ↓, decreased levels; FF, formula fed; BF, breastfed; CS, cesarean section; VD, vaginally delivered; PT, preterm; FT, full term; GOS, galacto-oligosaccharides; FOS, fructo-oligosaccharides*.

Although synbiotics, a combination of both a probiotic and a prebiotic (51), were not discussed in this review, the beneficial effects of bovine milk oligosaccharides and *Bifidobacterium* spp. on the infant gut have been noted in two human interventions (Table 1).

## Probiotic Intervention

### Health Benefits of Probiotics

Probiotics, described as “live microorganisms that, when administered in adequate amounts, confer a health benefit on the host” (52–55), have been investigated as potential prophylactics and/or treatments to re-establish gut homeostasis (Table 1). Moreover, the metabolism of indigestible oligosaccharides and plant polysaccharides by probiotic microorganisms, such as *Bifidobacterium* spp., contributes to the production of microbial bioactive molecules, such as SCFAs (56).

Subsequently, probiotic treatment is being extensively studied in different conditions associated with a disturbance in the gut. Necrotizing enterocolitis (NEC), childhood obesity and autism will be discussed next to highlight the link between a microbial disturbance in the gut and the beneficial use of probiotic prophylaxis in early life. The following conditions have been chosen due to their significant prevalence in current literature.

### Necrotizing Enterocolitis

Necrotizing enterocolitis, where portions of the bowel undergo necrosis, is the second most common cause of mortality in PT infants. Subsequent studies focused on improving health outcomes have found an increased abundance of Proteobacteria prior to and throughout the condition, including potentially pathogenic organisms such as *Salmonella* and *Escherichia coli* (57, 58). In a longitudinal study examining gut microbiota development in PT twins, a twin pair discordant for NEC was discovered. Results from this study found clear changes attributable to antibiotic exposure and NEC development, with reduced microbial diversity and an increase in *Escherichia* spp. preceding NEC (59).

Probiotic prophylaxis has thus been investigated to examine whether this form of treatment could improve the quality of life in PT infants. In a comprehensive review by AlFaleh and Anabrees (60), it was found that enteral administration of probiotics reduced incidence of severe NEC, and NEC-related mortality, with the majority of infants being administered a combination of *Lactobacillus, Bifidobacterium*, and *Streptococcus* spp. *via* breastmilk. Interestingly, the administration of bovine lactoferrin, in combination with *L. rhamnosus* GG, was found effective in reducing incidences of NEC in VLBW infants (61). In addition, the routine use of *Lactobacillus reuteri* DSM 17938 (BioGaia^®^) was found to be highly successful in reducing rates of NEC in infants at highest risk (birth weights ≤ 1,000 g) (62).

### Metabolic Syndrome (MS) and Obesity in Childhood

Metabolic syndrome, described by WHO in 1998, relates to any case of insulin resistance found in the presence of at least two of the following risk factors; hypertension, obesity, high triglyceride levels, or reduced high-density lipoprotein cholesterol levels. To examine the effectiveness of probiotics in preliminary animal trials against MS, *L. paracasei, L. rhamnosus*, and *Bifidobacterium animalis* subsp. *lactis* were found to improve glucose–insulin levels and hepatic steatosis in a high-fat diet (HFD)-induced murine model (63). However, in a systemic review on the use of early probiotic intervention in human clinical trials, inadequate evidence was found to support the use of the probiotic *L. rhamnosus* GG or *L. paracasei* F19, when administered to both mothers and infants, in the prevention of childhood MS (64).

To tackle the prevalence of childhood obesity, scientists have begun to unravel the link between diet, the gut microbiome and consequent energy intake and adiposity. Turnbaugh et al. (65) examined the hypothesis that particular communities of microorganisms could be involved directly with obesity in an obese mouse model. Results from the study found an increased capacity to harvest energy from diet, with an increased ratio of Firmicutes to Bacteroidetes (65). Further metagenomic analysis revealed that the microbiome of mice fed a high-fat/high-sugar (Western) diet, was significantly enriched in Kyoto Encyclopedia of Genes and Genomes (KEGG) pathways involved in the fermentation of simple sugars (66). Thus, with the aim of reducing HFD-induced weight gain in humans, animal studies are examining the antiobesity effects of different probiotics (67, 68). In-depth analysis of these animal studies may provide opportunities for the introduction of probiotics to help reduce weight gain in early life.

### Autism

The cause for autism spectrum disorder (ASD), a syndrome characterized by a deficit in social and communicative interactions, is yet unclear; however, recent studies have revealed a link between symptomatic cognitive dysfunctions and GI distress through a connection in the central nervous system, coining the term “brain–gut axis.” There is now evidence that probiotics alleviate GI distress in various murine models which mimic the symptomatic traits of ASD (69, 70). Studies have found that a member of the *Bacteroides* spp., *Bacteroides fragilis*, acts as a natural anti-inflammatory, capable of inhibiting inflammatory responses in a chemically induced murine model of experimental colitis (71, 72). Moreover, a maternal immune activation (MIA) model, which challenges the immune system and promotes inflammatory factors in pregnant dams, induces key features of ASD and thus serves as an appropriate murine model in testing *B. fragilis* as a potential therapeutic (70). Hsiao et al. (70) demonstrated the ability of *B. fragilis* to correct the levels of a MIA-induced serum metabolite which was found at significantly higher concentrations in MIA-offspring. Overall, *B.fragilis* improved gut permeability, as well as correcting ASD-related behavioral abnormalities. This suggests that a microbe-mediated therapy, such as *B. fragilis*, may alleviate various behavioral disorders during childhood.

## Conclusion

Throughout this mini-review, we have discussed the introduction of microbial therapeutics, in addition to prebiotic supplementation, to highlight the health benefits for their use in relieving GI distress in early life.

With regards to infant formulae, prebiotic supplementation with a mixture of GOS/FOS can help mimic the composition of breastmilk and promote the development of *Bifidobacterium* in the infant gut, in particular *B. longum*.

In terms of the clinical use of probiotics, it is crucial that we develop standardized treatments which take into account the age group of a specific human cohort, in addition to health status of the group in question. In this respect, the appropriate dose of a probiotic must be determined. Moreover, it is vital that we re-evaluate the safety of alternative probiotics, coined “next-generation probiotics” (NGPs). A preliminary evaluation on the safe use of a *Bacteroides xylanisolvens* isolate has recently been reported (73), in addition to the beneficial effects of *Faecalibacterium prausnitzii* (74, 75), and bacterial strains belonging to the *Eggerthellaceae* family, which produce metabolites with anti-inflammatory and cardioprotective properties (76). However, guidelines outlined by the European Food Safety Authority, and the Food and Agriculture Organization of the United Nations, have made it difficult to introduce these bacteria as food supplements. In terms of economic potential, further research is required to upscale these NGPs for food and/or pharmaceutical industries. The industrial challenges may be overcome through high throughput selection of bacterial strains with the capacity to grow well in selective media and tolerate the presence of oxygen. In other words, the technological robustness of the strain in question must be tested, in addition to the suitable anaerobic media and encapsulation methods required to retain probiotic viability under good manufacturing practices. Moreover, *in silico* screening of bacterial genomes will ensure the safety of these strains through the detection of antibiotic resistance and virulence genes. Alternatively, live biotherapeutic products (LBPs) may create an opportunity to introduce NGPs to the market (77). An LBP has recently been described as “a biological product that: (1) contains live organisms, such as bacteria; (2) is applicable to the prevention, treatment, or cure of a disease or condition of human beings; and (3) is not a vaccine” (78). In addition, O’Toole et al. (77) described future testing of LBPs as biological medicinal products, thereby providing new opportunities to introduce NGPs as well characterized drugs to the market.

Overall, we conclude that additional studies are necessary to investigate the influence of prebiotics and probiotics in early life. It is important that we consider the mixed microbial communities present within foods and select those which will survive and adapt readily in an industrial environment (79, 80). More importantly, we suggest if new probiotics and prebiotics are to be considered health beneficial in the European market, the necessity for comprehensive, randomized controlled trials is vital. The current approach requires further strategy to provide consumers with valid information toward the use of probiotic and prebiotic supplementation in early childhood.

## Author Contributions

RPR, CS, and CAR conceived the manuscript. CW drafted the manuscript. All authors reviewed the final version of the manuscript and approved it for publication.

## Conflict of Interest Statement

The authors declare that the research was conducted in the absence of any commercial or financial relationships that could be construed as a potential conflict of interest.

## Abbreviations

GI, gastrointestinal; FT, full term; PT, preterm; VLBW, very low birth weight; GOS, galacto-oligosaccharides; FOS, fructo-oligosaccharides; SCFAs, short chain fatty acids; NEC, necrotizing enterocolitis; MS, metabolic syndrome; HFD, high-fat diet; ASD, autism spectrum disorder; MIA, maternal immune activation; NGPs, next generation probiotics; LBPs, live biotherapeutic products.
